# Supplementary material for: Observation of anti-parity-time-symmetry, phase transitions and exceptional points in an optical fibre
Source: Nat Commun. 2021 Jan 20;12:486. doi: 10.1038/s41467-020-20797-7 (PMC7817694; doi:10.1038/s41467-020-20797-7)
Supplement: Supplementary file 1 — Supplementary information [file 41467_2020_20797_MOESM1_ESM.pdf]

Observation of anti-parity-time-symmetry, phase transitions and exceptional points in an optical fiber: Supplementary Information

Arik Bergman[1,2,*], Robert Duggan[1,3], Kavita Sharma[2], Moshe Tur[4], Avi Zadok[2,*] and Andrea Alù[1,3,5,*]

[1]*Photonics Initiative, Advanced Science Research Center, City University of New York, New York, New York 10031, USA*
[2]*Faculty of Engineering and Institute for Nano-Technology and Advanced Materials, Bar-Ilan University, Ramat-Gan 5290002, Israel*
[3]*Department of Electrical and Computer Engineering, University of Texas at Austin, Austin, Texas, 78712, USA*
[4]*School of Electrical Engineering, Tel-Aviv University, Tel-Aviv 6997801, Israel*
[5]*Physics Program, Graduate Center, City University of New York, New York, New York 10026, USA*
**Correspondence to be addressed to:* aalu@gc.cuny.edu; Avinoam.Zadok@biu.ac.il; Bergman.Arik@gmail.com

1. Derivation of the system Hamiltonian

Consider the optical fields $E_{1,2}$ of two continuous probe tones of frequencies $\nu_0 \pm \Delta\nu/2$ which co-propagate along the optical fiber under test (FUT)

$$E_{1,2}(r,z,t) = A_{1,2}(z)E_T(r) \times \exp[j(k_0 \pm \Delta k/2)z] \times \exp[-2\pi j(\nu_0 \pm \Delta\nu/2)t] + \mathrm{c.c.} \qquad (1)$$

Here r and z denote the radial and axial coordinates, respectively, t stands for time, and $\mathrm{c.c.}$ represents the complex conjugate. $E_T(r)$ (in units of m^{-1}) is the transverse profile of the optical mode, normalized so that $2\pi\int_0^\infty |E_T(r)|^2 r dr = 1$. The two waves co-propagate in the positive $\hat{z}$ direction with wavenumbers $k_0 \pm \Delta k/2$, where $k_0 = 2\pi(n/c)\nu_0$ and $\Delta k = 2\pi(n/c)\Delta\nu$. Here c is the speed of light in vacuum, and n represents the effective index of the single optical mode of a standard fiber. We assume that n is the same for all optical frequencies of interest. The two probe tones are co-polarized. The FUT is sufficiently short so that the evolution of polarization due to residual linear birefringence is neglected throughout the following analysis. We therefore describe the field components in scalar terms. Lastly, the complex magnitudes of the field components are noted by $A_{1,2}(z)$ (in units of Volts). The two magnitudes may change along the fiber due to backward stimulated Brillouin scattering (SBS) processes.

Consider also the optical fields of two continuous pump waves, counter-propagating in the negative $\hat{z}$ direction, at optical frequencies $\nu_0 + \nu_B \pm \Delta\nu/2$

$$E_{3,4}(r,z,t) = A_{3,4}E_T(r) \times \exp[-j(k_0 + k_B \pm \Delta k/2)z] \times \exp[-2\pi j(\nu_0 + \nu_B \pm \Delta\nu/2)t] + \mathrm{c.c.} \qquad (2)$$

Here ν_B is the Brillouin frequency shift (BFS) of the FUT, and $A_{3,4}$ denote the complex magnitudes of the pump tones. The BFS of standard single-mode fibers at 1550 nm wavelength is on the order of 11 GHz. The pump waves are taken to be in the undepleted regime, hence their magnitudes remain constant along the fiber. Linear losses along the short FUT are neglected as well. The wavenumber offset term k_B is defined as $k_B = 2\pi(n/c)\nu_B$. We assume that the states of polarization of both pump tones are aligned with that of the probe tones above.

SBS interactions between the pump and probe pairs generate longitudinal acoustic waves within the fiber, which co-propagate with the pump waves in the negative $\hat{z}$ direction. The frequencies of the stimulated acoustic waves are ν_B and $\nu_B \pm \Delta\nu$. Let us denote the density perturbations associated with the three acoustic wave components as $\Delta\rho_0$ and $\Delta\rho_{\pm 1}$, respectively

$$\Delta\rho_0(r,z,t) = B_0(z)u_T(r) \times \exp(-jq_0 z) \times \exp(-2\pi j\nu_B t) + \text{c.c.} \tag{3}$$

$$\Delta\rho_1(r,z,t) = B_1(z)u_T(r) \times \exp(-jq_0 z) \times \exp[-2\pi j(\nu_B + \Delta\nu)t] + \text{c.c.}$$

$$\Delta\rho_{-1}(r,z,t) = B_{-1}(z)u_T(r) \times \exp(-jq_0 z) \times \exp[-2\pi j(\nu_B - \Delta\nu)t] + \text{c.c.}$$

Here $q_0 = 2k_0 + k_B$ is the acoustic wavenumber, and $u_T(r)$ (in units of m^{-1}) is the transverse profile of the acoustic mode that is guided in the core of the fiber. The transverse profile is normalized so that $2\pi\int_0^\infty |u_T(r)|^2 r dr = 1$. Lastly, the complex magnitudes of the acoustic wave components (in units of kg×m^{-2}) are given by (*1*)

$$B_0(z) = j\frac{\varepsilon_0\gamma_e Q_{ES}}{2\pi\nu_B\Gamma_B} q_0^2[A_3A_1^*\exp(-j\Delta kz) + A_4A_2^*\exp(+j\Delta kz)] \tag{4}$$

$$B_1(z) = j\frac{\varepsilon_0\gamma_e Q_{ES}}{2\pi\nu_B\Gamma_B} q_0^2 A_3A_2^* \frac{1}{1-j\frac{2\pi\cdot\Delta\nu}{\Gamma_B/2}} = j\frac{\varepsilon_0\gamma_e Q_{ES}}{2\pi\nu_B\Gamma_B} q_0^2 A_3A_2^* \frac{1}{1-j\Delta}$$

$$B_{-1}(z) = j\frac{\varepsilon_0\gamma_e Q_{ES}}{2\pi\nu_B\Gamma_B} q_0^2 A_4A_1^* \frac{1}{1+j\frac{2\pi\cdot\Delta\nu}{\Gamma_B/2}} = j\frac{\varepsilon_0\gamma_e Q_{ES}}{2\pi\nu_B\Gamma_B} q_0^2 A_4A_1^* \frac{1}{1+j\Delta}.$$

In Eq. (4) $\Gamma_B \approx 2\pi \times 30$ MHz denotes the SBS linewidth in silica, ε_0 is the vacuum permittivity, γ_e is the electro-strictive constant of silica, and Q_{ES} denotes the spatial overlap integral between the transverse profiles of the electro-strictive driving force and the acoustic mode: $Q_{ES} = 2\pi\int_0^\infty |E_T(r)|^2 u_T^*(r) r dr$ (in units of m^{-1}). In the above expressions we have approximated $q_0 \pm \Delta k \approx q_0$. For brevity, we define $\Delta \equiv 2(2\pi\cdot\Delta\nu)/\Gamma_B$.

The interaction between the acoustic waves and the two pump tones introduces nonlinear polarization terms at the optical frequencies of the two probe waves, $\nu_0 \pm \Delta\nu/2$

$$P_1(r,z,t) = p_1(r,z) \times \exp[j(k_0 + \Delta k/2)z] \times \exp[-2\pi j(\nu_0 + \Delta\nu/2)t] + \text{c.c.} \tag{5}$$

$$P_2(r,z,t) = p_2(r,z) \times \exp[j(k_0 - \Delta k/2)z] \times \exp[-2\pi j(\nu_0 - \Delta\nu/2)t] + \text{c.c.}$$

with respective magnitudes

$$p_1(r,z) = \frac{\varepsilon_0\gamma_e}{\rho_0} E_T(r)u_T^*(r)[A_3B_0^*\exp(-j\Delta kz) + A_4B_{-1}^*(z)] \tag{6}$$

$$p_2(r,z) = \frac{\varepsilon_0\gamma_e}{\rho_0} E_T(r)u_T^*(r)[A_4B_0^*\exp(j\Delta kz) + A_3B_1^*(z)].$$

Here ρ_0 denotes the density of silica. The nonlinear polarizations may be substituted into the nonlinear wave equations of the two monochromatic probe waves

$$\nabla^2 E_{1,2}(r,z,t) + (k_0 \pm \Delta k/2)^2 E_{1,2}(r,z,t) = -\frac{[2\pi(\nu_0 \pm \Delta\nu/2)]^2}{\varepsilon_0 c^2} P_{1,2}(r,z,t). \tag{7}$$

Since probe fields $E_{1,2}(r,z,t)$ of constant magnitudes $A_{1,2}$ represent solutions of the homogeneous wave equation, all terms on the left-hand side which do not involve z-derivatives of $A_{1,2}(z)$ cancel out (*1*). We assume that both magnitudes are slowly varying, so that second derivatives of $A_{1,2}(z)$ may be disregarded (*1*). We also approximate $k_0 \pm \Delta k/2 \approx k_0$ and $\nu_0 \pm \Delta\nu/2 \approx \nu_0$. Next, we multiply both sides of the nonlinear wave equations by $E_T^*(r)$ and integrate over the transverse cross-section. Substituting Eq. (5) and Eq. (6), the pair of nonlinear wave equations take up the following form

$$2jk_0\frac{dA_1(z)}{dz} = -\frac{(2\pi\nu_0)^2}{c^2}\frac{\gamma_e}{\rho_0}Q_{ES}[A_3B_0^*\exp(-j\Delta kz) + A_4B_{-1}^*(z)] \tag{8}$$

$$2jk_0\frac{dA_2(z)}{dz} = -\frac{(2\pi\nu_0)^2}{c^2}\frac{\gamma_e}{\rho_0}Q_{ES}[A_4B_0^*\exp(j\Delta kz) + A_3B_1^*(z)].$$

Substituting from Eq. (4)

$$\frac{dA_1(z)}{dz} = \frac{\varepsilon_0\gamma_e^2Q_{ES}^2q_0^2\nu_0}{2n\rho_0c\nu_B\Gamma_B}\left[\left(|A_3|^2 + |A_4|^2\frac{1}{1-j\Delta}\right)A_1(z) + A_3A_4^*\exp(-2j\Delta kz)A_2(z)\right] \tag{9}$$

$$\frac{dA_2(z)}{dz} = \frac{\varepsilon_0\gamma_e^2Q_{ES}^2q_0^2\nu_0}{2n\rho_0c\nu_B\Gamma_B}\left[\left(|A_4|^2 + |A_3|^2\frac{1}{1+j\Delta}\right)A_2(z) + A_4A_3^*\exp(2j\Delta kz)A_1(z)\right].$$

The propagation of the two probe waves is therefore characterized by the SBS amplification of each tone by both pump waves, as well as coupling between the two. In our experiments, the two pump tones are of equal power levels P_p (in Watts), and of equal phases at their input end. We may therefore substitute $P_p = 2n\varepsilon_0c\left|A_{3,4}\right|^2 = 2n\varepsilon_0cA_3A_4^* = 2n\varepsilon_0cA_3^*A_4$

$$\frac{dA_1(z)}{dz} = \frac{\gamma_e^2Q_{ES}^2q_0^2\nu_0}{4n^2\rho_0c^2\nu_B\Gamma_B}P_p\left[\left(1 + \frac{1}{1-j\Delta}\right)A_1(z) + \exp(-2j\Delta kz)A_2(z)\right] \tag{10}$$

$$\frac{dA_2(z)}{dz} = \frac{\gamma_e^2Q_{ES}^2q_0^2\nu_0}{4n^2\rho_0c^2\nu_B\Gamma_B}P_p\left[\left(1 + \frac{1}{1+j\Delta}\right)A_2(z) + \exp(2j\Delta kz)A_1(z)\right].$$

Next, we may define the SBS gain coefficient of the fiber, in units of $W^{-1}\times m^{-1}$

$$\gamma \equiv \frac{\gamma_e^2Q_{ES}^2q_0^2\nu_0}{2n^2\rho_0c^2\nu_B\Gamma_B}. \tag{11}$$

The power gain in standard SBS processes, in which a single probe wave is amplified by a single pump wave and the two are detuned by the BFS ν_B, is given by $\exp(\gamma P_pz)$. The value of γ in standard single-mode fibers at 1550 nm wavelength is on the order of 0.1-0.2 $W^{-1}\times m^{-1}$. Using this definition, the coupled wave equations of the probe tones may be written more concisely

$$\frac{dA_1(z)}{dz} = \frac{\gamma}{2}P_p\left[\left(1 + \frac{1}{1-j\Delta}\right)A_1(z) + \exp(-2j\Delta kz)A_2(z)\right] \tag{12}$$

$$\frac{dA_2(z)}{dz} = \frac{\gamma}{2}P_p\left[\left(1 + \frac{1}{1+j\Delta}\right)A_2(z) + \exp(2j\Delta kz)A_1(z)\right].$$

The equations may be processed further using a change of variables, $A_{1,2}(z) = a_{1,2}(z)\exp(\mp j\Delta kz)$

$$\frac{da_1(z)}{dz} = j\Delta k\cdot a_1(z) + \frac{\gamma}{2}P_p\left[\left(1 + \frac{1}{1-j\Delta}\right)a_1(z) + a_2(z)\right] \tag{13}$$

$$\frac{da_2(z)}{dz} = -j\Delta k\cdot a_2(z) + \frac{\gamma}{2}P_p\left[\left(1 + \frac{1}{1+j\Delta}\right)a_2(z) + a_1(z)\right],$$

leading to the following representation

$$j\frac{d}{dz}\begin{bmatrix}a_1(z)\\a_2(z)\end{bmatrix} = \begin{bmatrix}-\Delta k + j\frac{\gamma}{2}P_p\left(1 + \frac{1}{1-j\Delta}\right) & j\frac{\gamma}{2}P_p\\ j\frac{\gamma}{2}P_p & \Delta k + j\frac{\gamma}{2}P_p\left(1 + \frac{1}{1+j\Delta}\right)\end{bmatrix}\begin{bmatrix}a_1(z)\\a_2(z)\end{bmatrix} \equiv \mathcal{H}_0\begin{bmatrix}a_1(z)\\a_2(z)\end{bmatrix}. \tag{14}$$

Equation (14) defines the Hamiltonian of the probe waves propagation. In our experiments $\Delta \ll 1$, and we may approximate

$$\mathcal{H}_0 \approx \begin{bmatrix} -\left(\Delta k + \frac{\gamma}{2} P_p \Delta\right) + j\gamma P_p & j\frac{\gamma}{2} P_p \\ j\frac{\gamma}{2} P_p & \left(\Delta k + \frac{\gamma}{2} P_p \Delta\right) + j\gamma P_p \end{bmatrix}. \tag{15}$$

This form also appears in the main text. The eigen-values of the system are given by

$$\sigma_{\pm} = \frac{1}{2}\mathrm{trace}(\mathcal{H}_0) \pm \sqrt{\left[\frac{1}{2}\mathrm{trace}(\mathcal{H}_0)\right]^2 - \det(\mathcal{H}_0)} = j\gamma P_p \pm \sqrt{(\Delta k + \frac{\gamma}{2} P_p \Delta)^2 - (\frac{\gamma}{2} P_p)^2}. \tag{16}$$

The exceptional point is reached when the two eigen-values above coalesce into one. When $\Delta \approx 0$, this condition is met when the following simple relation is maintained between the pump power and probe waves detuning

$$\Delta k = \frac{\gamma}{2} P_p. \tag{17}$$

The required frequency detuning between the pair of probe tones is therefore given by

$$\Delta\nu_{EP} = \frac{c}{4\pi n}\gamma P_p. \tag{18}$$

For $\Delta \neq 0$, the exceptional point condition becomes

$$\widetilde{\Delta k} = \frac{\gamma}{2} P_p, \tag{19}$$

with a modified wavenumber mismatch term:

$$\widetilde{\Delta k} = \Delta k + \frac{\gamma}{2} P_p \Delta. \tag{20}$$

The frequency detuning at the exceptional point in this case equals

$$\Delta\nu_{EP} = \frac{1}{4\pi} \times \frac{\gamma P_p}{\frac{n}{c} + \gamma P_p \frac{1}{\Gamma_B}}. \tag{21}$$

For the experimental conditions used in this work, Δ is on the order of few percent, and the correction for $\Delta\nu$ at the exceptional point is of similar proportion. Higher-order corrections to the eigen-values solutions are not necessary. When the frequency detuning $\Delta\nu$ is larger than $\Delta\nu_{EP}$, the two eigen-values have equal imaginary parts, and their real parts differ. The opposite holds below $\Delta\nu_{EP}$, where the real parts of $\sigma_{\pm}$ are the same and the imaginary parts become different.

The eigen-vectors corresponding to $\sigma_{\pm}$ are given by

$$\vec{e}_{\pm} = \begin{bmatrix} j\dfrac{\widetilde{\Delta k} \mp \sqrt{\widetilde{\Delta k}^2 - (\frac{\gamma}{2} P_p)^2}}{\frac{\gamma}{2} P_p} \\ 1 \end{bmatrix}. \tag{22}$$

2. Deviations from the Brillouin frequency shift

Suppose next that the frequency separation between the upper (lower) pump tone and the upper (lower) probe tone does not exactly match the BFS ν_B, but differs from the BFS by a small-scale $\Delta\nu_B$. We assume that the deviation is much smaller than the Brillouin linewidth: $\Delta\nu_B \ll \Gamma_B$, and define a

normalized offset parameter $\Delta_B \equiv 2(2\pi \cdot \Delta\nu_B)/\Gamma_B$. Repeating the derivation of the previous section, we reach a modified Hamiltonian for the propagation of the two probe tones

$$\mathcal{H} = \begin{bmatrix} -\Delta k + j\frac{\gamma}{2}P_p\left(\frac{1}{1+j\Delta_B} + \frac{1}{1-j\Delta+j\Delta_B}\right) & j\frac{\gamma}{2}P_p\frac{1}{1+j\Delta_B} \\ j\frac{\gamma}{2}P_p\frac{1}{1+j\Delta_B} & \Delta k + j\frac{\gamma}{2}P_p\left(\frac{1}{1+j\Delta_B} + \frac{1}{1+j\Delta+j\Delta_B}\right) \end{bmatrix}. \quad (23)$$

At the limit of $\Delta, \Delta_B \ll 1$, we may approximate the Hamiltonian in terms of a first-order perturbation correction

$$\mathcal{H} \approx \mathcal{H}_0 + \frac{\gamma}{2}P_p\Delta_B \begin{bmatrix} 2 & 1 \\ 1 & 2 \end{bmatrix}. \quad (24)$$

The eigen-values of the modified Hamiltonian are approximately given by

$$\sigma_\pm \approx j\gamma P_p + \gamma P_p\Delta_B \pm \sqrt{(\Delta k + \frac{\gamma}{2}P_p\Delta)^2 - \left(\frac{\gamma}{2}P_p\right)^2 + j2\Delta_B\left(\frac{\gamma}{2}P_p\right)^2}. \quad (25)$$

The mean of the two eigen-values and their difference are modified by Δ_B. With $\Delta_B \neq 0$, the system cannot be brought to an exceptional point with pump tones of equal power. When the pump power P_p and probe waves detuning $\Delta\nu$ are adjusted to the exceptional point conditions of $\mathcal{H}_0$ (Eq. (19)), the splitting between the two eigen-values scales with the square root of Δ_B

$$\Delta\sigma(\Delta\nu_B) \equiv (\sigma_+ - \sigma_-)/2 \approx \sqrt{j/2}\sqrt{\Delta_B}\gamma P_p. \quad (26)$$

The square-root dependence of the eigen-values splitting in the vicinity of the exceptional point suggests an enhanced response to small-scale variations in the BFS. Note that the splitting from the exceptional point affects both the real and imaginary parts of the eigen-values.

The eigen-vectors of the modified Hamiltonian are given by

$$\vec{e}_\pm \approx \begin{bmatrix} j\dfrac{\widetilde{\Delta k} \mp \sqrt{\widetilde{\Delta k}^2 - (\frac{\gamma}{2}P_p)^2 + j2\Delta_B\left(\frac{\gamma}{2}P_p\right)^2}}{\frac{\gamma}{2}P_p(1-j\Delta_B)} \\ 1 \end{bmatrix}. \quad (27)$$

References

1. R. W. Boyd, *Nonlinear Optics*, 3rd Edition, (Academic, 2008).
